# Quantitative evaluation of anti-resorptive agent-related osteonecrosis of the jaw using bone single photon emission computed tomography in clinical settings: relationship between clinical stage and imaging

**DOI:** 10.1007/s12149-020-01485-4

**Published:** 2020-06-15

**Authors:** Taro Okui, Yoshikazu Kobayashi, Masakazu Tsujimoto, Koji Satoh, Hiroshi Toyama, Koichiro Matsuo

**Affiliations:** 1 Department of Dentistry and Oral-Maxillofacial Surgery, School of Medicine, Fujita, Japan; 2 Department of Radiology, Fujita Health University Hospital, 1-98 Dengakugakubo, Kutsukake, Toyoake, Aichi 4701192 Japan; 3 Department of Radiology, School of Medicine, Fujita Health University, 1-98 Dengakugakubo, Kutsukake, Toyoake, Aichi 4701192 Japan

**Keywords:** Anti-resorptive agent-related osteonecrosis of the jaw, Bone SPECT/CT, Metabolic bone volume

## Abstract

**Objective:**

This study aimed to use quantitative values, calculated from bone single photon emission computed tomography (SPECT) imaging, to estimate the reliability of progression evaluation for anti-resorptive agent-related osteonecrosis of the jaw (ARONJ).

**Methods:**

The study population consisted of 21 patients (23 lesions), clinically diagnosed with mandibular ARONJ, who underwent SPECT/CT scanning. Diagnosis and staging of ARONJ were performed according to the American Association of Oral and Maxillofacial Surgeons (AAOMS) definition and the recommendations of the International Task Force on ONJ. Hybrid SPECT/CT imaging quantitative analyses were performed on a workstation. Each volume of interest (VOI) was semi-automatically placed over a lesion with areas of high tracer accumulation, using the GI-BONE^®^ software default threshold method settings. Additionally, control VOI was manually set over an unaffected area. Measured parameters included standardized uptake values (SUV)—maximum (SUV_max_) and mean (SUV_mean_), metabolic bone volume (MBV)—the total volume above the threshold, and total bone uptake (TBU) as calculated by MBV × SUV_mean_. We also calculated the SUV ratio (rSUV) between the lesion and control area, factoring for differences in individual bone metabolism; the ratios were termed rSUV_max_ and rSUV_mean_, accordingly. The product of multiplying the rSUV_mean_ by MBV of a lesion was defined as the ratio of TBU (rTBU). Quantitative values were compared between clinical stages by the Kruskal–Wallis test and subsequent post hoc analysis.

**Results:**

MBVs (cm^3^) were: median, [IQR] Stage 1, 8.28 [5.62–9.49]; Stage 2, 15.28 [10.64–24.78]; and Stage 3, 34.61 [29.50–40.78]. MBV tended to increase with stage increase. Furthermore, only MBV showed a significant difference between clinical stages (*p *< 0.01). Subsequent post hoc analysis showed no significant difference between stages 1 and 2 (*p *= 0.12) but a significant difference between stages 2 and 3 (*p *= 0.048). rSUVmax and rTBU tended to increase with stage increase, but the differences between the stages were not significant (*p* = 0.10 and *p* = 0.055, respectively).

**Conclusion:**

MBV, which includes the concept of volume, showed significant differences between clinical stages and tended to increase with the stage increase. As an objective and reliable indicator, MBV might be an adjunct diagnostic method for staging ARONJ.

## Introduction

Administration of anti-resorptive agents is a standard treatment for bone metastases of malignancy, multiple myeloma, or osteoporosis [1]. Since the first report by Marx [2] on necrosis of the jaws that was induced by drug injection, the number of articles on anti-resorptive agent-related osteonecrosis of the jaw (ARONJ) has continuously increased. ARONJ is classified into three stages according to the American Association of Oral and Maxillofacial Surgeons (AAOMS) definition [3] and the recommendations of the International Task Force on ONJ position paper [4]. The stages are defined based on the presence of infection as determined by clinical findings, and by the extent of the disease as determined by radiographs. The differences between stages indicate differences in treatment strategies, with conservative treatment recommended for Stage1 and surgical treatment for stages 2 and 3 [4]. However, as the criteria for staging of ARONJ involve many judgment items and subjective factors, the staging of the same patient may often differ between treating professionals.

The usefulness of bone scintigraphy in the diagnosis of ARONJ is well established. Bone scintigraphy can demonstrate the anatomy of bony structures and also detect diseases that alter the metabolic activity of the bone without any structural or anatomical alteration, thereby enabling the detection of early status disease that has not yet produced any structural changes [5].

The hybrid single photon emission computed tomography (SPECT), in combination with a CT system (SPECT/CT), provides more diagnostic certainty and anatomical localization than the conventional bone scanning for ARONJ. Such a system combines the simultaneous assessment of morphological changes on CT images with functional changes in bone turnover on SPECT images [6]. Furthermore, the development of bone SPECT quantitative analysis software has facilitated the calculation of the standardized uptake value (SUV) and lesion volume from bone SPECT images. Such calculations are used to evaluate the effect of treatment on bone metastases of malignant tumors [7].

The ability to evaluate ARONJ using quantitative values obtained from bone SPECT/CT images would provide a more objective and reliable index for intra- and inter-individual comparisons. This approach, when used as an adjunct to conventional staging methods, could be useful for treatment planning.

The purpose of this study was to investigate whether quantitative values, calculated from bone SPECT images, could be used to assess the severity and progression of ARONJ. We also aimed to identify which quantitative values would be most predictive. To achieve these objectives, we compared the quantitative values obtained from the bone SPECT images at different clinical stages.

## Materials and methods

### Patients

Between April 2017 and December 2018, we continuously enrolled participants who were diagnosed with mandibular ARONJ at Fujita Health University Hospital and for whom the imaging data that were acquired included panoramic radiographs, CT images, and bone SPECT/CT of the head and neck regions. The population consisted of 21 patients (23 lesions). This observational study was conducted in accordance with the Declaration of Helsinki 1964, and was approved by the Institutional Ethics Committee of Fujita Health University (Reference number: 17-170). In 9 of the 21 patients, scans were performed prospectively. These patients were fully briefed on the purpose of this study, and their written informed consent was obtained. Data from the other patients were retrospectively reviewed. These patients were given a lengthy opt-out period (from September 2017 to March 2020) and information that the data would be investigated anonymously was published on the Fujita Health University Hospital website.

### Image interpretation and definition of ARONJ staging

The diagnosis and staging of ARONJ were performed according to the American Association of Oral and Maxillofacial Surgeons (AAOMS) definitions [3] and the recommendations of the International Task Force on ONJ [4]. The diagnostic criteria for ARONJ were as follows: (1) exposed bone in the maxillofacial region that does not heal within 8 weeks after identification by a healthcare provider; (2) exposure to an anti-resorptive agent; and (3) no history of radiation therapy to the craniofacial region.

The clinical stages, based on the AAOMS definitions and ARONJ International Task Force on the Osteonecrosis of the Jaw, were as follows: Patients with Stage 1 disease have exposed bone, but are asymptomatic and show no evidence of significant adjacent or regional soft-tissue inflammation or infection. Stage 2 disease is characterized by exposed bone with associated pain, and adjacent or regional soft-tissue inflammatory swelling or secondary infection. Stage 3 disease is characterized by exposed bone with associated pain, and adjacent or regional soft-tissue inflammatory swelling or secondary infection, as in Stage 2. In addition, patients with Stage 3 disease would have a pathologic fracture, an extraoral or oral–antral fistula, or radiographic evidence of osteolysis extending to the inferior border of the mandible or the floor of the maxillary sinus. Diagnosing and staging were based on clinical findings, panoramic radiographs, and CT images performed during the first visit, and a discussion among three oral and maxillofacial surgeons.

### Bone SPECT/CT

Hybrid SPECT/CT imaging of the head and neck regions was performed using Symbia T6 (Siemens K.K., Tokyo, Japan), which combines dual-head gamma camera with a multi-slice spiral CT scanner. Data were acquired by performing whole-body planar and SPECT/CT imaging 3 h after intravenous injection of 740 MBq of ^99m^Tc-HMDP. Acquisition conditions of SPECT images were as follows: matrix size of 128 × 128, pixel size of 3.90 × 3.90 × 3.90 mm^3^, slice thickness of 3.90 mm, enlargement by 1.23-times, and the main energy window set to 140 keV ± 7.5%. The sub energy window for scatter component estimation was set to 7% lower than 140 keV. The number of views was 64 over 360°, in continuous mode, while the orbit was non-circular. SPECT image reconstruction was performed using Flash 3D [8] in a reconstruction algorithm. The number of subsets was eight, the number of iterations was 18, and the Gaussian filter was set to 8 mm [9]. Low-dose CT scans of the head and neck regions were performed as follows: tube voltage of 130 kV, tube current of 50 mAs, and matrix size of 512 × 512. For CT image reconstruction, slice thickness was set to 3.0 mm, and the reconstruction increment was 1.5 mm. B08s SPECTAC was used in the reconstruction function. Images were sent directly to a bundled workstation and fused manually.

### Image analysis and quantification

**Fig. 1:**
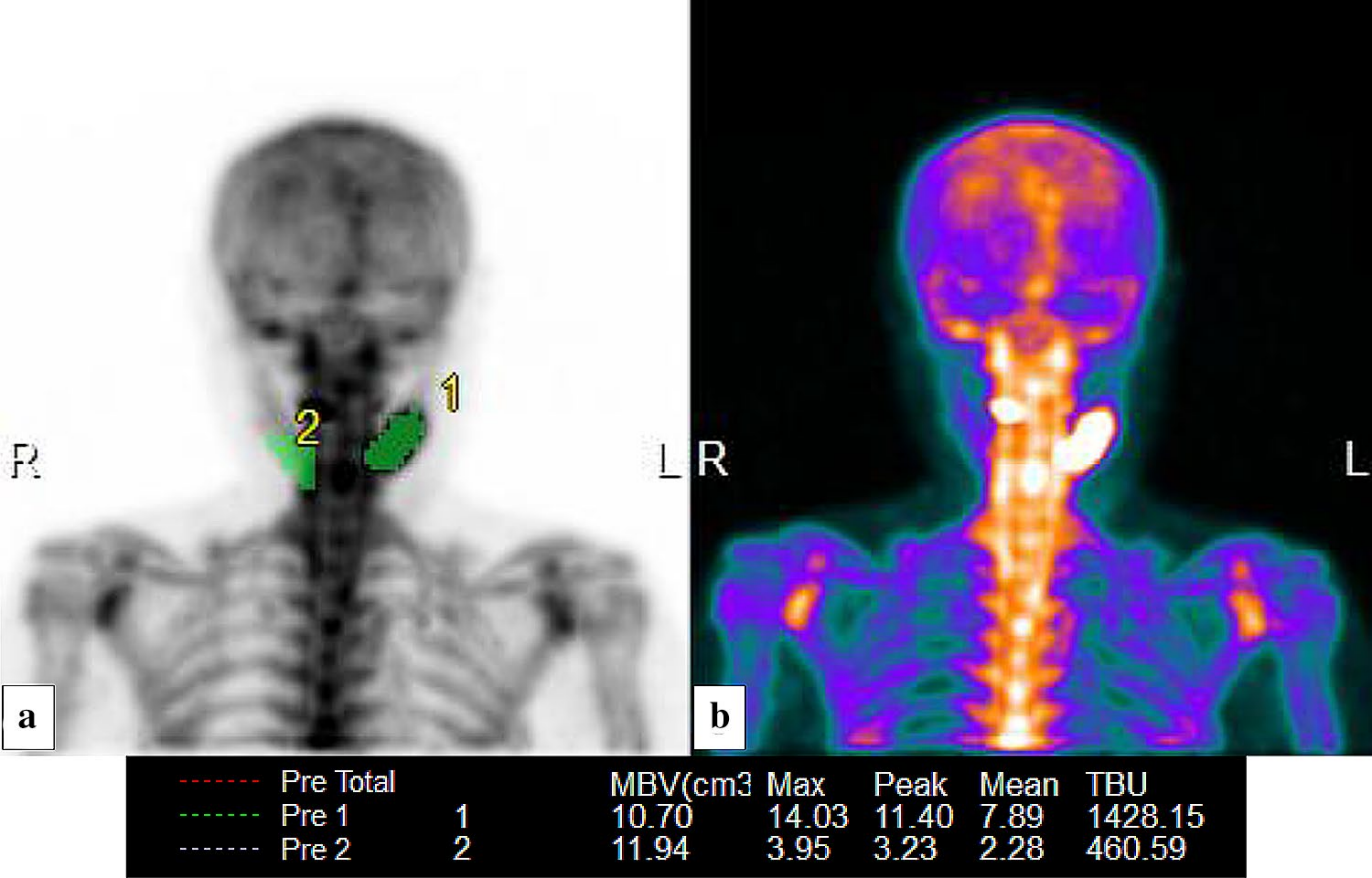
Volume of interest setting on the GI-BONE^®^ software. **a** Detection result of lesion with high accumulation and unaffected control region and **b** original image. The VOI of a lesion (1) in the left mandible was semi-automatically placed over a lesion with an area of high tracer accumulation using the threshold method available within the software. The threshold value was automatically calculated by obtaining a histogram from the target range and adding 0.5 standard deviation (SD) to the base value. This base value was obtained as the pixel value reduced from the most frequent value to 95%. The VOI in an unaffected area (2) was manually set within a mandibular bone region without tracer accumulation. MBV, SUV_max_, SUV_peak_, SUV_mean_, and TBU values were automatically calculated by the GI-BONE^®^ software. The calculated quantitative values are shown below the images. The SUV_peak_ value was not examined in this study. MBV, metabolic bone volume; SUV_max_, SUV_peak_ and SUV_mean_, maximum, peak, and mean standardized uptake values, respectively; *TBU* total bone uptake, *VOI* volume of interest

Image analysis was performed using the Virtual Place Hayabusa and GI-BONE^®^ tumor analysis package (Nippon Medi-Physics, Tokyo, Japan). Each volume of interest (VOI) was manually placed over a lesion with an area of high tracer accumulation, using the threshold method. The threshold was determined based on a previously reported method [10]. This method is available within the software. The threshold value was automatically calculated by obtaining a histogram from the target range and adding 0.5 standard deviation (SD) to the base value. This base value was obtained as the pixel value reduced from the most frequent value to 95%. Additionally, control VOI was manually set over an unaffected area in the contralateral side. In patients with bilateral lesions, the control VOI was set in the center of the mandible (Fig. 1). Decision on the unaffected area was made after referencing the CT images and following discussion among two nuclear medicine radiologists and two oral and maxillofacial surgeons. For quantitative assessment, the standardized uptake value (SUV) was calculated using the following formula:

$${\text{SUV}} = \frac{{{\text{pixel value of SPECT}}\;\left[ {{\text{Bq}}/{\text{mL}}} \right]}}{{{\text{Radioactivity administered}}\; \left[ {\text{Bq}} \right]/{\text{body weight}}\;\left[ g \right]}} \times 100\left[ \% \right].$$

Measured or calculated parameters were SUV_max_, SUV_mean_, Metabolic Bone Volume (MBV) - total volume above the threshold, and Total Bone Uptake (TBU) as calculated by MBV × SUV_mean_. We also calculated the SUV ratio (rSUV) between the lesion and control area, factoring for the difference between individual bone metabolisms. The ratio calculated by dividing the SUV_max_ of the lesion by the SUV_max_ of the unaffected area was thus termed rSUV_max_, and the ratio calculated by dividing the SUV_mean_ of the lesion by the SUV_mean_ of the unaffected area was thus termed rSUV_mean_. The product of multiplying rSUV_mean_ by MBV of a lesion was defined as the ratio of TBU (rTBU).

### Statistical analysis

SPECT quantitative values were compared between clinical stages using the Kruskal–Wallis test, and subsequent post hoc analysis using the Steel–Dwass test. Differences were considered statistically significant when *p* < 0.05. Statistical analyses were performed using EZR ver. 1.38 (available at http://www.jichi.ac.jp/saitama-sct/SaitamaHP.files/statmedEN.html).

## Results

**Fig. 2:**
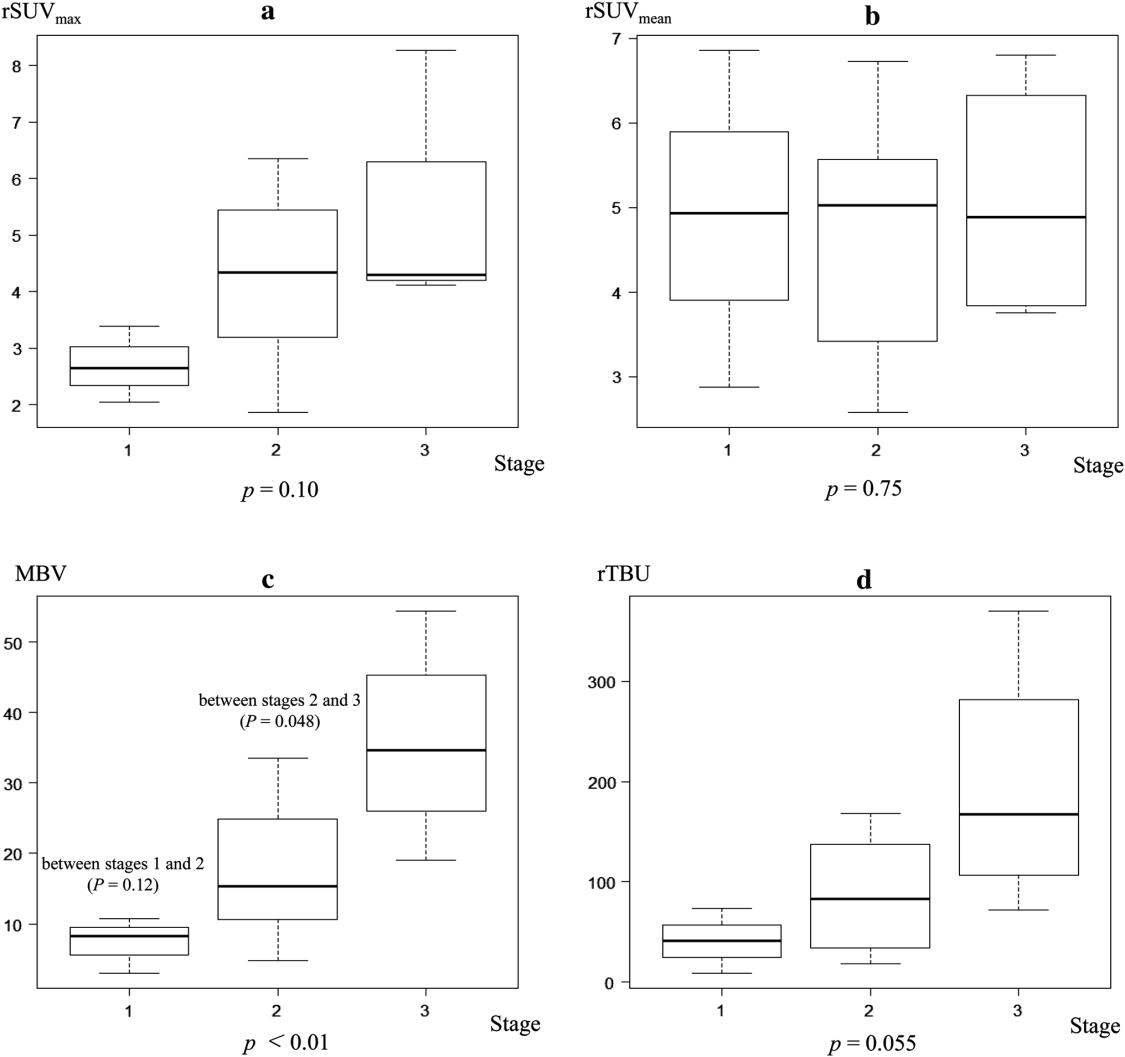
Quantitative SPECT bone uptake values with respect to clinical stages. **a** rSUV_max_, **b** rSUV_mean_, **c** MBV, and **d** TBU. MBV tended to increase with increasing stage of disease. Only MBV showed a significant difference between clinical stages (*P* < 0.01). Results of the subsequent post hoc analysis showed no significant difference between stages 1 and 2 (*P* = 0.12), but a significant difference between stages 2 and 3 (*P* = 0.048). The quantitative values of rSUV_max_ and rTBU tended to increase with the increasing stage of disease, but differences between the stages did not reach the significance level (*P* = 0.10 and *P* = 0.055, respectively). rSUV_mean_ did not differ between clinical stages (*P* = 0.75). *MBV* metabolic bone volume; rSUV_max_ and rSUV_mean_, ratios of maximum and mean standardized uptake values, respectively; rTBU, the product of rSUV_mean_ multiplied by MBV; SPECT, single photon emission computed tomography

Patients’ characteristics, including sex, age, type of anti-resorptive agent, target illness, and stage of ARONJ, are shown in Table 1. Of the 23 lesions, three were at Stage 1, 16 at Stage 2, and four at Stage 3. The results of the quantitative analysis are presented as boxplots in Fig. 2. Values for rSUV_max_ were: median, [IQR] Stage1, 2.65 [2.35–3.02]; Stage2, 4.34 [3.28–5.43]; Stage3, 4.30 [4.24–5.31]. Differences between the stages were insignificant (*P *= 0.10). Values for rSUV_mean_ were: median, [IQR]Stage1, 4.93 [3.90–5.90]; Stage2, 5.03 [3.44–5.50]; Stage3, 4.89 [3.88 - 6.09]. Differences between the stages were insignificant (*P *= 0.75). Values for MBV(cm^3^) were: median, [IQR]Stage1, 8.28 [5.62–9.49]; Stage2, 15.28 [10.64– 24.78]; Stage3, 34.61 [29.50–40.78]. There were significant differences between stages (*P *< 0.01). Values for rTBU were: median, [IQR] Stage1. 40.85 [24.68–57.15]; Stage2, 82.85 [35.33–135.65]; Stage3, 167.52 [124.40–237.36]. Differences between the stages were insignificant (*P *= 0.055). MBV tended to increase with increasing stage of disease. Results of the subsequent post hoc analysis showed no significant difference between stages 1 and 2 (*P *= 0.12), but a significant difference between stages 2 and 3 (*P *= 0.048). The quantitative values of rSUV_max_ and rTBU tended to increase with the increasing stage, but the differences between the stages did not reach the significance level.

**Table 1:** Patients’ characteristics

Patient	Lesion	Sex	Age	Type of anti-resorptive agent	Target illness	Stage
1	1	M	84	Zoledronate → denosumab	Prostate cancer	3
2	2	F	77	Minodronate	Primary osteoporosis	2
3	3	M	87	Denosumab	Prostate cancer	2
4	4	F	78	Minodronate	Primary osteoporosis	2
5	5 6	M	73	Zoledronate	Lung cancer	3 1
6	7	F	63	Denosumab	Lung cancer	2
7	8	F	67	Alendronate	Glucocorticoid-induced osteoporosis	2
8	9 10	F	68	Zoledronate → denosumab	Breast cancer	2 1
9	11	F	68	Zoledronate → Risedronate → minodronate	Glucocorticoid-induced osteoporosis	2
10	12	F	68	Zelodronate	Breast cancer	2
11	13	F	79	Alendronate → Risedronate → Ibandronate	Glucocorticoid-induced osteoporosis	2
12	14	M	80	Zoledronate → Denosumab	Prostate cancer	2
13	15	F	83	Zoledronate → Denosumab	Primary osteoporosis	1
14	16	F	72	Denosumab	Breast cancer	2
15	17	M	71	Denosumab	Lung cancer	2
16	18	F	55	Zelodronate	Multiple myeloma	2
17	19	M	72	Denosumab	Prostate cancer	3
18	20	F	67	Denosumab	Breast cancer	3
19	21	F	69	Zoledronate → denosumab	Breast cancer	2
20	22	F	79	Alendronate	Glucocorticoid-induced osteoporosis	2
21	23	F	58	Denosumab	Breast cancer	2

## Discussion

In this study, we investigated the differences between clinical stages in each of several quantitative values calculated from bone SPECT images. Only MBV, a parameter based on the concept of volume, showed a significant difference between the clinical stages and tended to increase with increasing stage of disease. For management of ARONJ that is associated with secondary infection, surgical interventions are often indicated [11]. In those cases, sufficient debridement and resection of the affected bone are necessary to prevent a recurrence. However, it is important to evaluate the affected lesion and preserve regeneratable bone to prevent postoperative functional disorders.

ARONJ is usually diagnosed based on clinical manifestations and history of anti-resorptive agents’ administration [3]. Plain radiography, CT, and MRI are used both to diagnose and to assess progress of ARONJ [12]. These imaging modalities are useful to visualize bony changes and evaluate the involvement of adjacent structures, such as the mandibular canal or maxillary sinus [13]. While these modalities can be used to visualize the lesions after structural alterations, they cannot detect early changes in ARONJ or inflammatory activities [5]. ARONJ is a complex of various pathological conditions. The extent of bone exposure is in disaccord with progression of inflammation. It is, therefore, difficult to identify the range of the lesion before surgery, and setting the resection margins might depend on the intraoperative subjective judgment of the operators.

Hybrid SPECT/CT systems combine nuclear tomographic imaging with low-dose CT scanning to provide practical information and visualize chronic osteomyelitis and bone metabolic activity before any structural or anatomical changes become apparent. Such systems might significantly increase the accuracy of anatomical localization and facilitate diagnosis as well as treatment planning [6]. Recently, the development of bone SPECT quantitative analysis software has enabled the calculation of SUV and lesion volume from bone SPECT images [7]. However, the usefulness of bone SPECT quantitative values for clinical status assessment and the correlation between uptake values and clinical stage have not been well discussed.

**Fig. 3:**
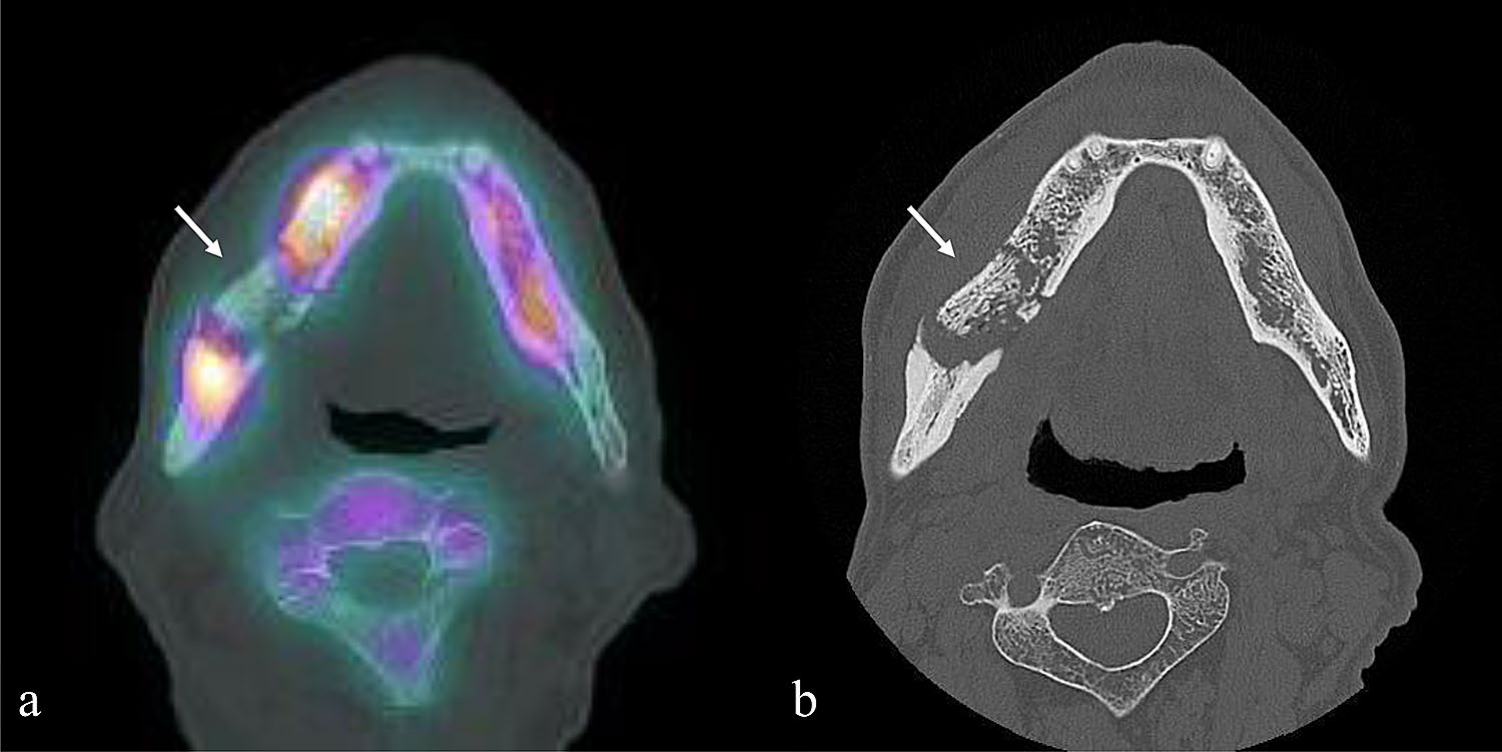
Findings of a “cold-in-hot” region. **a** SPECT/CT image showing an involucrum with low accumulation surrounded by high-accumulation area on the right mandible (see arrow). **b** CT image showing that the involucrum is separated from the surrounding bone. *CT* computed tomography, *SPECT* single photon emission computed tomography

**Fig. 4:**
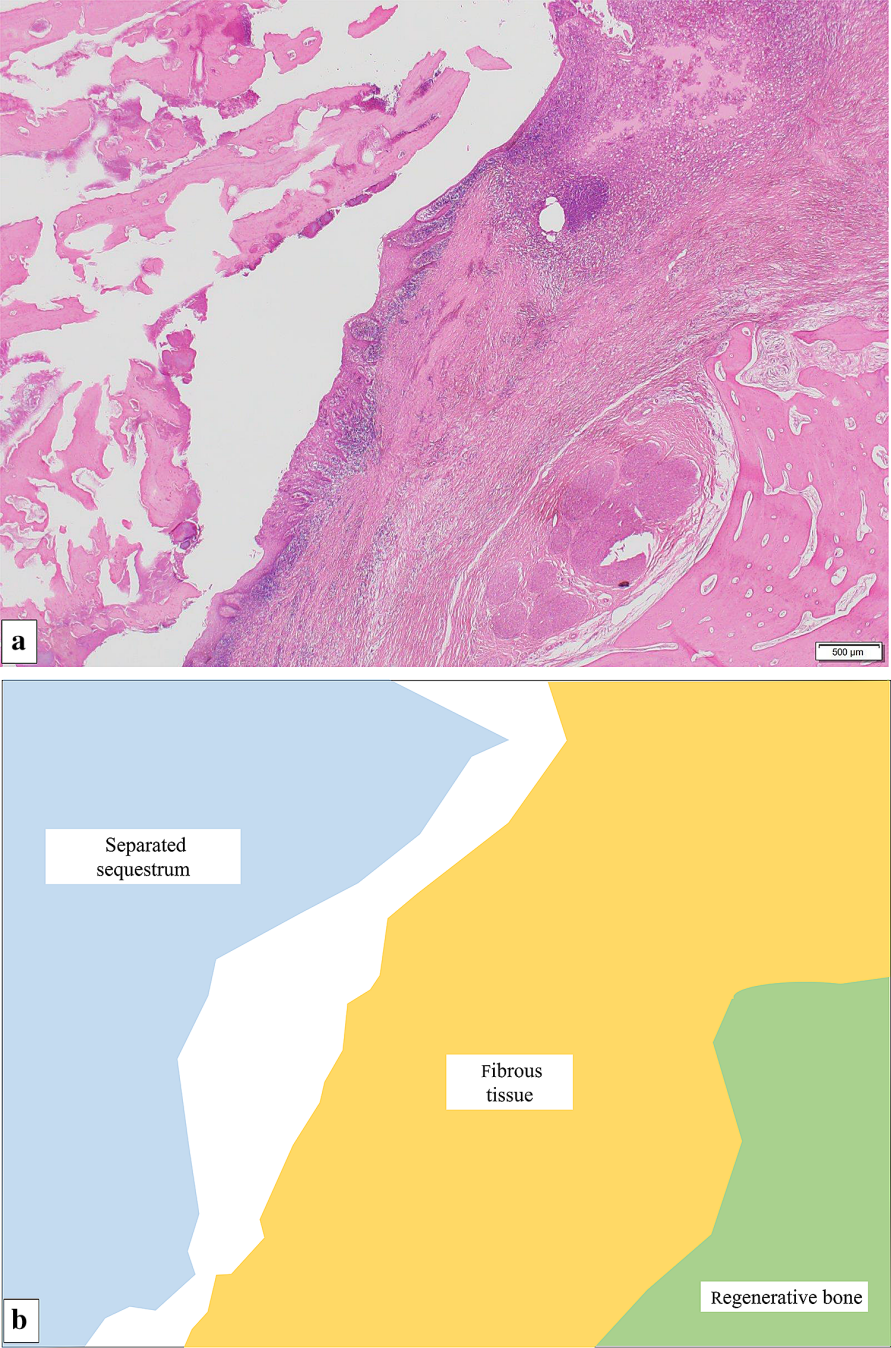
Pathological findings in a “cold-in-hot” region from the area indicated by arrow in Fig. 3. **a** Histopathology and **b** schematic drawing of a “cold-in-hot” region. On the left, a “cold area” is present with separated sequestrum. On the right, a “hot area” is seen as a hypermetabolic region that includes fibrous tissue and regenerative bone

In this study, we investigated the differences between clinical stages for each quantitative value calculated from bone SPECT images. Anti-resorptive agents might accumulate not only in the affected lesion, but also through the rest of the body, including the surrounding healthy mandibular bone. To address this issue, we calculated the SUVs ratio between the lesion and healthy parts of the bone to calibrate individual bone metabolic activity. While some differences were noted in rSUV_max_ and rTBU, only MBV showed a significant difference, whereas rSUV_mean_ showed no difference between clinical stages. The reason differences between clinical stages could not be observed in rSUV_mean_ might be because, in the lesions where the sequestrum was separated from the surrounding bone following their progression, the radioactive tracer could not reach the lesions. This is because of the diminished vascularity, which creates the so-called “cold-in-hot” regions (Fig. 3). Each “cold-in-hot” area consists of a “cold area” with sequestrum and a hypermetabolic “hot area,” including fibrous tissue and pathological regenerative bone (Fig. 4). Differences between stages, however, were meaningful in MBV, a parameter based on the concept of volume.

The staging system is particularly valuable for ARONJ treatment strategy optimization. The AAOMS recommends conservative treatment (administration of antimicrobial medication and local irrigation) for Stage1 of ARONJ, and surgical treatment for stages 2 and 3. The staging system proposed by the AAOMS is based on the presence of infection and complications, and the depth into which the lesion has progressed [3]. Because high-stage ARONJ is associated with inflammation arising from infections or complications, the staging of ARONJ would benefit from a combination of bone scintigraphy to assess the severity of inflammation at specific sites [5], clinical assessment of symptoms of nonspecific infection, and imaging studies.

In the process of staging mandibular ARONJ, we estimate the severity and the progress of the disease and search for severe complications, such as neuropathy or pathologic fractures, which are caused mainly by the vertical progression of the lesion. In the evaluation of imaging studies, the mandibular canal and the inferior border of the mandible are important anatomical landmarks, assessed to determine the vertical progression of the disease. In a Position Paper of the Japanese Allied Committee on Osteonecrosis of the Jaw from 2017, ARONJ Stage3 was defined based on the horizontal progression in the mandibular ramus [14]. In determining the excision border for mandibular ARONJ, imaging studies are useful in the horizontal plane. In the vertical plane, marginal or segmental resection is selected, depending on whether the lesion extends to the mandibular canal or not. Since high-stage lesions progress vertically and horizontally, it was thought, and the study has confirmed, that only MBV would show a significant difference between clinical stages. Furthermore, in the post hoc analysis of MBV, no difference was found between stages 1 and 2, but a significant difference was found between stages 2 and 3. The reason might be that in ARONJ staging, the evaluation criterion for classifying stages 1 and 2 is the presence of secondary infection, while the concept of volume is ignored. In contrast, stages 2 and 3 are classified according to the concept of volume. As MBV includes the concept of volume, it showed a significant difference between these advanced clinical stages and tended to increase with the increasing stage of disease. This suggests that a high-accumulation region in the SPECT might indicate the advancing ARONJ. As an objective and reliable indicator, MBV might, therefore, be an adjunct diagnostic method for staging ARONJ.

This study has some limitations. First, there were a few patients in stages 1 and 3, while the majority were in Stage2. Second, the total number of patients included in the study was relatively small, thereby limiting the statistical power to detect significant associations. A prospective study, with a large number of subjects, is warranted to verify the usefulness of bone SPECT quantitative values as tools to assess the severity and progression of ARONJ. A major limitation of this study is the lack of comparison with CT imaging or with pathological findings. Diagnostic CT imaging that allows evaluation of bone microstructural changes. In the present study, all four patients with Stage3 disease showed bone structural changes of lysis and sclerosis in the high-accumulation area of SPECT/CT. If compared to CT images, some correlation may be obtained. However, this study was not designed to compare diagnostic CT images and SPECT findings and does not have sufficient data to do so. It is, thus, impossible to assess such a correlation. Future studies comparing the accumulation spread on SPECT/CT with lesion progression on CT images are needed.

Currently, histopathological findings are considered to be the gold standard for differentiating between the various disease states of ARONJ. Histopathological examination can identify necrosis, inflammation, fibrosis, and more, which are all affected by local vascularity. This study has shown that SPECT/CT images and MBV, a quantitative value obtained from these images, are useful for assessing the severity and progression of ARONJ. To confirm whether the increased uptake predominantly indicates bone destruction or osteogenesis, it is necessary to compare SPECT/CT images and MBV with histopathological findings. We have started collecting such information from cases that require surgery for future analysis. The Stage3 case shown in Figs. 3, 4 were a representative case in which the SPECT/CT imaging findings could be compared with the CT imaging findings and the pathological findings.

Finally, the method of threshold setting used in this study was the software’s default settings. Previous studies referenced also did not reveal any related evidence. To establish an appropriate threshold, future studies are needed to compare SPECT/CT findings with CT findings and histopathology.

In conclusion, MBV, which includes the concept of volume, showed a significant difference between clinical stages, and tended to increase with increasing stage of disease. This suggests that the high-accumulation regions in SPECT images might indicate the advancing ARONJ. As an objective and reliable indicator, MBV might be an adjunct diagnostic method for staging ARONJ.
