# Correction to: Quantitative evaluation of anti-resorptive agent-related osteonecrosis of the jaw using bone single photon emission computed tomography in clinical settings: relationship between clinical stage and imaging

**DOI:** 10.1007/s12149-022-01755-3

**Published:** 2022-05-18

**Authors:** Taro Okui, Yoshikazu Kobayashi, Masakazu Tsujimoto, Koji Satoh, Hiroshi Toyama, Koichiro Matsuo

**Affiliations:** 1 Department of Dentistry and Oral-Maxillofacial Surgery, School of Medicine, Fujita, Japan; 2 Department of Radiology, Fujita Health University Hospital, 1-98 Dengakugakubo, Kutsukake, Toyoake, Aichi 4701192 Japan; 3 Department of Radiology, School of Medicine, Fujita Health University, 1-98 Dengakugakubo, Kutsukake, Toyoake, Aichi 4701192 Japan

## Correction to: Annals of Nuclear Medicine (2020) 34:620–628 10.1007/s12149-020-01485-4

The article “Quantitative evaluation of anti-resorptive agent-related osteonecrosis of the jaw using bone single photon emission computed tomography in clinical settings: relationship between clinical stage and imaging”, written by Taro Okui, Yoshikazu Kobayashi, Masakazu Tsujimoto, Koji Satoh, Hiroshi Toyama, Koichiro Matsuo, was originally published Online First without Open Access. After publication in volume 34, issue 9, page 620–628, the author decided to opt for Open Choice and to make the article an Open Access publication. Therefore, the copyright of the article has been changed to © The Authors 2022 and the article is forthwith distributed under the terms of the Creative Commons Attribution 4.0 International License, which permits use, sharing, adaptation, distribution and reproduction in any medium or format, as long as you give appropriate credit to the original author(s) and the source, provide a link to the Creative Commons licence, and indicate if changes were made.

The images or other third party material in this article are included in the article’s Creative Commons licence, unless indicated otherwise in a credit line to the material. If material is not included in the article’s Creative Commons licence and your intended use is not permitted by statutory regulation or exceeds the permitted use, you will need to obtain permission directly from the copyright holder.

To view a copy of this licence, visit http://creativecommons.org/licenses/by/4.0/.

